# Effect of 4 weeks of frankincense consumption on explicit motor memory and serum BDNF in elderly men

**DOI:** 10.3906/sag-1810-204

**Published:** 2019-08-08

**Authors:** Elham ASADI, Mohammad Reza SHAHABI KASEB, Rasool ZEIDABADI, Mohammad Reza HAMEDINIA

**Affiliations:** 1 Department of Motor Behavior, Faculty of Sport Sciences, Hakim Sabzevari University, Sabzevar Iran; 2 Department of Sport Physiology, Faculty of Sport Sciences, Hakim Sabzevari University, Sabzevar Iran

**Keywords:** Frankincense, explicit motor memory, BDNF, elderly

## Abstract

**Background/aim:**

Memory is a mechanism for coding, storing, and recalling information. Weak memory and learning disability are common psychological problems in the elderly. The aim of this study was to investigate the effect of 4 weeks of frankincense consumption on explicit motor memory and serum BDNF in the elderly.

**Materials and methods:**

Twenty elderly men (mean age of 60.2 ± 1.7 years) were randomly divided into two groups: experimental (n = 12) and placebo (n = 8). The first blood samples were collected 24 h before the pretest. Then both groups participated in a 4-week exercise program based on the protocol of exercising motor memory. During this period, the experimental group received 500-mg frankincense pills two times a day. The second blood sample collection and acquisition test were conducted following the last session of the exercise program. A retention test and a third blood sampling were performed 2 weeks after the last training session. Mixed analysis of variance (2 × 3) for repeated measures was used to analyze the data.

**Results:**

Intergroup comparisons showed that frankincense had a significant effect on the acquisition and retention of explicit motor memory. No difference was observed in serum BDNF between the experimental and placebo groups.

**Conclusion:**

This study revealed that 4 weeks of frankincense consumption facilitates the acquisition and retention of motor memory in older men with moderate mental status.

## 1. Introduction

Learning and memory are among the highest functional levels of the central nervous system. Learning is the process by which we get information about the world around us. However, memory is a mechanism for encoding, storing, and recalling previously learned information [1]. Researchers have divided memory into two parts: short-term and long-term memory. Storage of information in short-term memory is generally limited in terms of capacity and duration. With repetition and mental review, this information is transmitted from short-term memory to long-term memory [2]. Long-term memory is formed when special neuronal connections have been permanently reinforced. Long-term memory is categorized into two groups, based on the information type: explicit and implicit memory. The explicit memory is related to past events with conscious awareness, while implicit memory is related to past events without conscious awareness [3]. Explicit motor memory is a type of explicit memory. The explicit motor memory is responsible for movements ranging from skeletal movements to language movements, which can be learned through practice and experience [4]. Weakness of this kind of memory impairs acquisition of motor sequences, which often learns without the person’s attention for learning and only through sequential implementation of a repetitive motor pattern [5].

Aging is a part of the biological process of all living beings, including humans [6]. The improved health care services of today have led to increased life expectancy, in turn increasing the world’s aging population [7]. The number of elderly people is expected to increase to 152 million by 2050 and the aging rate will reach 37.5% [8]. Among the diseases whose prevalence increases with age are neurodegenerative diseases, such as cognitive impairment [9,10]. Cognitive impairment is a form of disease that can disrupt attention, memory, and performance. It damages the memory region of the brain in the elderly [11].

Memory impairment is a common result of the aging process and can be a sign of Alzheimer disease and dementia [7]. As age increases, the reduction in the size of the prefrontal gyrus of the dorsal-external and hippocampus areas disrupts the formation, preservation, and recall memory performance as well as cognitive function [12–15]. Studies have shown that reduction in hippocampal volume has a direct correlation with reduction in brain-derived neurotrophic factor (BDNF) [16,17]. BDNF is a secretion protein with 119 amino acids; there is a high level of BDNF in the brain, especially in the hippocampus [18]. This factor modifies a variety of actions, including neurological survival, neurogenesis, cell death, axonal growth, conjunction, and plasticity [19].

BDNF contributes greatly to nervous plasticity and is effective throughout life in empowering essential functions such as learning and memory [18]. Different studies have shown a possible relationship between low levels of BDNF and conditions such as depression, neurodegenerative disorders, Alzheimer disease, and dementia [19,20]. Studies have shown that the use of chemical and biological drugs has a positive effect on the cognitive memory function of the elderly [21] but intake of this type of drug has resulted in unpleasant side effects in many patients who were looking for other options for treating cognitive problems [22]. With new advances in chemistry and pharmacy, the use of alternative treatments—especially herbal treatment—is increasing [21,23]. Ginger, lemon balm, and frankincense are among the herbal medicines used in treating the elderly, especially people with amnesia [7,24].

In Iran, frankincense was used by the famous physician Avicenna to prevent amnesia and improve memory in the 10th century [25]. The most important component of frankincense is its resinous part and the main ingredients are incensole acetate and boswellic acid. According to previous studies, frankincense can have a positive effect on brain development, and possibly on the formation of dendrites and axons and improving their communication [24]. The side effects of frankincense in humans are very rare and can be disregarded. Furthermore, no adverse interaction of frankincense with other medications has been reported [26]. Previous studies have confirmed improved memory due to the consumption of frankincense in animals [21,25,27,28]. On the other hand, studies in humans suggested that the use of frankincense improved general memory in the elderly [29,30], spatial memory [31], cancer and chronic inflammatory diseases, brain abnormalities, and memory disorders [32].

As can be seen, most of the studies focused on the effects of frankincense consumption on cognitive memory in animals [33,34] or patients with cognitive impairment [31]. There has been an increase in the population of elderly people in Iran [35] and a lack of scientific research on the effects of herbal medicine on elderly motor memory. Therefore, the purpose of the present study was to investigate the effect of 4 weeks of frankincense consumption on explicit motor memory and serum BDNF in the elderly.

## 2. Materials and methods

### 2.1. Participants

This was an applied and semiexperimental study with a pretest–posttest design and a placebo group. The sample population consisted of elderly men in the city of Sabzevar. First, the volunteers completed the individual profile form. The Edinburgh Handedness Inventory and Mini-Mental State Examination (MMSE) questionnaires were then distributed among the volunteers. After the forms were reviewed, 20 eligible volunteers (right-handed, moderate mental status) were selected. The participants were able to follow the instructions correctly. The reason for choosing participants with moderate mental status was so as not to include samples with Alzheimer disease or superior memory. After receipt of the filled-in consent forms, the participants were randomly divided into a treatment group (12 participants) and a control group (8 participants). This research was registered at the Clinical Trials Base with the receipt of the code of ethics (IR.IAU.S.REC.1396.8) from the Deputy Assistant Professor of Research and Technology at the Islamic Azad University of Sabzevar and all participants provided informed consent in the format required by the relevant authorities.

### 2.2. Materials

The Edinburgh Handedness Inventory Questionnaire was used to assess the dominant (preferred) hand of each person. This tool is a 10-item questionnaire that assesses the dominance of a person’s left or right hand with the help of the following: writing, drawing, throwing, using scissors, brushing teeth, using a knife, using a spoon, using a broom, lighting a match, and opening and closing the lid of a can. This test has five options, where an individual scores two for performing the task always with the right hand, with both hands, and always with the left hand, and one for performing the task often with the right and left hands. The total score ranges from −100 (left) to +100 (right). Cronbach’s alpha was 0.97 and its correlation was 0.92 [36].

The Mini-Mental State Examination (MMSE) questionnaire was divided into six areas: orientation, registration, attention and calculation, recall, language functions, and visuospatial thinking. This test consisted of three items—each with 5 points—and lasted about 30 min. Each item had five questions with 1 point. The total score of this test was between 0 and 30, with 0 to 10 signifying cognitive intense disorder, 11 to 20 signifying intermediate disorder, 21 to 26 signifying cognitive slight disorder, and 27 to 30 signifying normalcy. Cronbach’s alpha for the test was 0.81 [37].

The linear move machine model (LM – 01) tool was used for motor memory measurement, to measure the amount of displacement linear motion of the upper limb. The machine resembled a measured piece of wood with a handle on a pipe. The participant’s eyes were closed and he moved the handle several times to a marked obstacle, placed 30 cm from the starting point. The participant was then asked to remember and cover the distance he had created with the hand without any obstacle. The LM machine records the distance that person covers in millimeters. The tool’s reliability was 0.90 and Cronbach’s alpha was 0.93 [38].

Frankincense was analyzed phytochemically. The chemical content of frankincense includes 25% to 35% insoluble gum in alcohol, 60% to 70% resin, and the remaining is a kind of essence. Boswellic acids and incensole acetate are the main ingredients of frankincense [39]. Among the important derivatives of boswellic acids may be β-boswellic acid (BA), 11-keto-B-boswellic acid (KBA), 3-acetyl β-boswellic acid (ABA), and 3-acetyl-11 keto β-boswellic acid (AKBA) [40]. Frankincense was acquired from the medicinal plant research center of Barij (Kashan, Iran) (batch number: BS10017). The amounts of total boswellic acid, KBA, and AKBA were estimated using a calibration curve [41].

The laboratory kits for evaluation of BDNF were obtained from the East Biopharm Company in Italy.

### 2.3. Procedure

The first blood sample was taken between 0700 and 0900. The pretest was conducted in the laboratory after 10 h of fasting in order to evaluate the serum BDNF levels. The participants’ lab motor behavior was assessed. They first learned to work with the machine and then nine trials were conducted to make them familiar with the machine, so that they could handle it for a specified distance (30 cm) with their eyes closed. In each of the three trials, the average error feedback (distance from the marker to the criterion distance) was presented. Subsequently, each participant’s performance in the pretest—which included a triple block with a nondominant hand, closed eyes, and absolute value average error, conducted in three trials—was calculated and recorded. The treatment and placebo groups consumed two doses of 500-mg capsules of frankincense and chickpea flour, respectively. They took the doses daily after two meals in the morning and evening for 4 weeks. Participants from both groups were told to practice the motor task with the nondominant hand and closed eyes for 4 weeks, with two sessions each week (three blocks of four). After each block, average feedback was given to the participants. During these 4 weeks, the elderly participants were reminded about daily consumption of the capsules. The second blood sample was collected after the acquisition test (after the last training session), in the same manner that the first blood sample was collected in. A third blood sample was collected and a retention test was performed 2 weeks after the last session, where the participants neither consumed the capsules nor did they practice anything. Acquisition and retention tests consisted of three trials with the nondominant hand without vision. During the blood sampling, blood samples (5 cc) were collected from a vein in special tubes (BD Vacutainer® SST II Advance) and remained at the usual temperature for 20 min to clot and after centrifugation (15 min to 3000 rpm) the serum was frozen at –80 °C. The serum BDNF was measured by ELISA using a special kit (R&D BDNF ELISA kit, Italy) with double repetition and the use of an ELISA reader (Awareness Technology, Palm City, FL, USA). (The range of measuring BDNF kits from 8/7 to 500 picograms to milliliters).

### 2.4. Statistical analysis

For descriptive statistics, the mean and standard deviation were used in the present study. Moreover, the Kolmogorov–Smirnov and Levene tests were used for data normalization and homogeneity of variances, respectively. For statistical analysis of the data, mixed variance analysis (2 × 3), post hoc test, repeated measures, the nonparametric Friedman test, and the Mann–Whitney U test were performed at the probability level of P = 0.05, using SPSS 22.

## 3. Results

The descriptive results of the data of the two groups are presented in Table 1.

As shown in Table 1, the mean error of the treatment group in the acquisition and retention test is less than that in the placebo group.

As shown in Table 2, the effect of time in the treatment and placebo groups is significant (P = 0.006), but the interactive effect of time × group is not statistically significant (P > 0.98).

The post hoc test of repeated measures for the treatment and placebo groups showed that the effect of time in the treatment group was significant (P < 0.05). However, the same on the placebo group was not significant (P > 0.05). Further reviews and results of the Bonferroni post hoc test, with Bonferroni moderation (α/2 = 0.025), showed that there was a significant difference between the pretest and the acquisition test (P < 0.025). However, no significant difference was found between the acquisition test and the retention test (P > 0.025).

According to Table 3, the difference between the groups was significant in acquisition and retention (P < 0. 016). It should be noted that for significant analysis in this section, Bonferroni moderation (α/3 = 0.016) was used.

Due to the nonnormalization of BDNF data, nonparametric tests were used to scrutinize the same. The results of the two groups in the pretest were compared, which showed that there was no significant difference between the groups (P > 0.05). Furthermore, the results of the Friedman test showed that there was no significant difference between the stages of the pretest and acquisition and retention tests in either group (P > 0.05).

According to Table 4, there was no significant difference between the treatment and placebo groups in the acquisition or retention tests.

## 4. Discussion

The purpose of this study was to review the effect of 4 weeks of frankincense consumption on explicit motor memory and serum BDNF in elderly men as far as acquisition and retention were concerned.

### 4.1. Effect of 4 weeks of frankincense consumption on acquisition and retention of motor memory in the elderly

The results of this study showed that 4 weeks of frankincense consumption was effective on explicit motor memory of the elderly in the acquisition stage. The results of the present study are presented in this section along with other results [33,34] that showed that 4 weeks of frankincense consumption was effective on spatial memory acquisition in rats. According to the other results [42], 8 weeks of frankincense consumption had a significant effect on the spatial memory acquisition of rats (P = 0.03). In other research [27] it was reported that frankincense extract consumption for 2 weeks was effective on spatial memory acquisition in rats. It appeared that frankincense influenced the sensitive areas of learning, especially the hippocampus. This, in turn, increased the message transmission and memory enhancement [43]. This was due to changes in the synaptic conductivity and the formation of new synaptic networks and increased synaptic power. Moreover, the results of this study in this section were consistent and showed that [25] 8 weeks of frankincense consumption improved the acquisition of spatial memory in older rats. In fact, laboratory studies showed that the main ingredient of frankincense, boswellic acid, could increase dendritic shoots in hippocampal neurons [43]. It could also prevent the instability of microtubule proteins as a factor in the degeneration of the neurons in the elderly [44]. In other words, other results showed that the combination of two herbs—frankincense and lemon balm—for 1 month in the elderly (without Alzheimer disease) improved general memory and two subscales: immediate and auditory memory [29]. Moreover, 8 weeks of frankincense consumption by patients with MS improved their spatial memory acquisition [31]. Research showed that protein kinase played an important role in the processing of memory [45,46]. Therefore, it could be assumed that the effect of frankincense on memory is due to the interaction of boswellic acid with the kinase protein pathways. On the other hand, the mechanism by which frankincense had a positive effect on memory might be due to the expansion of interactions with inflammation mediators and neurotransmitters [28].

In addition, incensole acetate and boswellic acid were the main ingredients of frankincense and had antiinflammatory effects. These could decrease the nervous degeneration by affecting the brain [47]. Laboratory studies using animal models suggested that the boswellic acid caused inhibition of the inflammatory prefactors [26]. In other words, immunohistochemistry studies showed that patients with memory disorder had inflammation in certain areas of the brain. Prescribed antiinflammatory drugs prevented memory loss in these patients. Therefore, it could be concluded that a part of the effect of frankincense on memory improvement was due to its antiinflammatory effects [48]. On the other hand, frankincense was a known memory enhancer and it prevented Alzheimer disease [49]. According to pharmacological studies, frankincense had materials that helped maintain memory [50]. Moreover, the results of the present study showed that 4 weeks of frankincense consumption affected the retention (2 weeks) of motor memory in the elderly. The results of the present study in this section were compared with other results [42], which stated that the effect of 8 weeks of frankincense consumption on retention (P = 0.02) of the spatial memory of rats was significant. This effect was probably established through inhibition of the phosphodiesterase enzyme and prevention of the presynaptic membrane CAMP hydrolysis involved in the memory and learning process. That was similar to the effect of serotonin on the sensitizing phenomenon [51]. It appeared that frankincense could enhance the proliferation of axons with reflection circuits and cause stability in them and the total of these actions results in memory improvement.

### 4.2. Effect of 4 weeks of frankincense consumption on serum BDNF in the posttest and delayed test (2 weeks)

The results in this section showed that 4 weeks of frankincense consumption was not effective on serum BDNF in the posttest and delayed test (2 weeks). The results of this study contradicted the other results, according to which, 4 weeks of frankincense consumption in adult rats increased BDNF [34]. One of the reasons for the contradictory results of the two studies was the measurement method for BDNF. Because of the animal sample, it was possible to measure BDNF through the tissue of the hippocampus. However, in the present study, BDNF was measured by blood sampling. In other words, specific levels of BDNF could vary from day to day due to the amount of cortisol or hormonal changes. Moreover, the energy balance might partially affect the concentration of BDNF in the environment [52].

No effects of frankincense on BDNF level were observed in the present study. This might be due to the dependency on the duration of frankincense consumption, which in a human sample 4 weeks of frankincense consumption may not be able to show its influence on the BDNF at blood levels. On the other hand, we were not able to measure the amount of BDNF in hippocampus tissue.

On the other hand, low levels of BDNF are associated with malfunctioning cognitive learning, depression, and conditions for nerve degradation [53,54], and research has shown that there is a relationship between increased aerobic exercise in the volume of the hippocampus with increasing BDNF levels [55]. In the present study, there were no differences in BDNF level, probably due to the lack of examination of aerobic exercise and depression in the elderly men.

It is recommended that more studies focus on changes in the duration of frankincense consumption, how it is consumed, and the consideration of individual differences to investigate the effect of frankincense on the motor memory of the elderly.

Based on the results of the present study, we can say that 4 weeks of frankincense consumption at a dose of 1000 mg per day facilitates motor memory in elderly men with moderate mental status in terms of acquisition and retention.

## Acknowledgments

We sincerely thank the honorable professors of Hakim Sabzevari University, as well as Dr Faramarzi, Dr Sezavar Labs, and the Sabzevar Retirement Center, for helping us in this research.
